# Sufficient blood, safe blood: can we have both?

**DOI:** 10.1186/1741-7015-10-29

**Published:** 2012-03-23

**Authors:** Halvard Bönig, Michael Schmidt, Kai Hourfar, Jörg Schüttrumpf, Erhard Seifried

**Affiliations:** 1 German Red Cross Blood Service Baden-Württemberg-Hesse and Institute for Transfusion Medicine and Immunohematology of the Goethe University, Frankfurt, Germany

**Keywords:** blood safety, blood consumption, MSM, donor deferral, donor retention, donor recruitment

## Abstract

The decision in September 2011 in the UK to accept blood donations from non-practicing men who have sex with men (MSM) has received significant public attention. Will this rule change substantially boost the number of blood donations or will it make our blood less safe? Clearly, most European countries have a blood procurement problem. Fewer young people are donating, while the population is aging and more invasive therapies are requiring more blood. Yet if that was the reason for allowing non-practicing MSM to donate, clearly re-admission of some other, much larger populations that are currently deferred from donation should likewise be considered. As far as risks for blood safety are concerned, evidence has been provided that the current quality of infectious disease marker testing significantly mitigates against, although does not completely eradicate, risks associated with admission of donors with a high risk of carrying certain blood-transmissible agents. However, it could be argued that more effective recruitment of the non-donor pool, which is substantially larger than the group of currently ineligible donors, would be a better strategy. Recruitment of this group will benefit the availability of blood without jeopardizing the current excellent safety profile of blood.

## Background

Demand for blood is ever-increasing as continuous improvements in medicine have ensured that people live longer, and thus will cumulatively receive more (blood-consuming) medical treatment. No adequate synthetic or bio-engineered alternatives for blood, that is, no pharmaceuticals which might replace lost oxygen carrying capacity or primary hemostasis, have been developed. In contrast to most other pharmaceuticals, therefore, blood comes from a very poorly controlled source: volunteer donors. Blood donors are exposed to all kinds of environmental factors, which are, at least in theory, transmissible with the donated blood, including infectious agents and pharmaceuticals.

To ensure blood safety, a long list of anamnestic exclusion criteria has accumulated [1]. This includes individuals who have recently undergone medical interventions potentially associated with bacteremia or viral infection, those who have travelled to places with increased risk for certain infectious agents, people who take certain medications, and also those whose behavior predisposes them to a higher risk for blood-born infections, such as men who have sex with men (MSM). In this way, scores of volunteers who would be willing to donate blood are deferred, many of them indefinitely. In an increasingly risk-averse society such as ours, new exclusion criteria are defined, while existing ones are rarely challenged. However, it is becoming difficult to satisfy the increasing need for blood in an aging population where the younger generation is notoriously hard to motivate to donate blood.

In September 2011, one exclusion criterion was repealed in the UK in response to pressure from special interest groups. This decision caused quite a splash: "Men who have sex with other men will be allowed to donate blood" reads a BBC News headline [2]. Hailed or cursed as the lift of the ban of MSM from blood donation in Britain by gay-rights-advocates or opponents, the rule does nothing more than relax donor eligibility rules to include MSM who have abstained from homosexual intercourse for ≥ 12 months. This is far from the revolution it has been perceived to be by the public on both sides of the fence. This commentary will discuss if or how this change will affect blood supply or (perceived) blood safety. Moreover, we will briefly report on prospected blood use vs. blood availability, and provide a short paragraph on how currently the infectious safety of blood is ascertained.

### Blood donor eligibility

"Men who have sex with other men will be allowed to donate blood" [2]. How big an issue is the change in donor eligibility rules? Reliable numbers about how many men engage, or have ever engaged, in sexual intercourse with men are hard to ascertain. Consistently, a frequency in excess of 5% of all men has been reported [3]. With > 50% of all newly diagnosed cases of HIV within their population, MSM indisputably remain the most significant risk population [4].

That said, predicting how the change in donor eligibility rules in Britain will affect blood donor behavior and blood safety is not straight-forward. For starters, it is impossible to estimate how many individuals are affected by the rule change. We neither know how many abstinent MSM there are nor how many of them aspire to becoming regular blood donors. At the same time, it is known that sexually active MSM were not necessarily deterred by the ban on blood donation, but that some of them rather donated quite regularly, to obtain free and anonymous HIV screening [5]. For the past decade, with few exceptions, all first-time HIV serology or nucleic acid testing (NAT) positive blood donors diagnosed at our institution admitted to being MSM, having withheld their sexual orientation upon previous donations (unpublished data). Even though donors from risk populations are requested to suggest disposal of their donor blood, with rare exceptions, those men had not. Thus the blood of many infectious disease marker (IDM)-negative MSM has been unwittingly transfused; however, thanks to sensitive screening technology, most of the potential harm was averted. With this in mind, admission of non-practicing MSM, whose risk at being in the window period of currently used diagnostic tests should be the same as in the general population if they flocked to the blood drives, might even increase rather than decrease the safety of our blood supply, by diluting the contribution of practicing (high-risk) MSM. Realistically, however, dramatic quantitative or qualitative consequences of the "British revolution" for the blood supply must not be expected [5].

### How secure is our blood supply?

The two tasks of the transfusion medicine community - optimal blood safety and a steady supply of blood - are mutually exclusive if taken to the extreme. Clearly, modern medicine, in spite of blood-saving technologies, requires more and more blood. A multitude of reasons or this has been mentioned, the most important being ever more aggressive, blood-consuming therapies and an aging population whose prolonged biological youthfulness extends their eligibility for novel, highly invasive treatments recently reviewed in [6]]. At the same time, blood is becoming an increasingly scarce commodity: The young population is shrinking, as is their willingness to donate blood. Public support of the idea of blood donation as a public service is dwindling, and the federal government continues to tighten eligibility criteria. A dramatic disparity of supply and demand of blood products is imminent [6].

Activities, such as implementation of optimal blood use programs, development of evidence-based indications for blood use and "optimized donation management" can reduce blood consumption and waste and thus slow the growth of blood use, and the recent increase of maximum donor age has provided temporary relief [7,8]. However, the question on how to meet future demand, even if it did not increase, remains unanswered.

After the recent act of allowing non-practicing MSM to donate blood, we should pause to ask whether some reasons for donor exclusion might not be comparatively esoteric, and whether their lifting might actually have some tangible benefit for the availability of blood without entailing measurable recipient risks. Creutzfeld Jacob's disease New Variant (vCJD) may be a good example. At the peak of the Bovine Spongiform Encephalopathy (BSE) crisis 10 to 15 years ago, the cumulative world-wide annual incidence of vCJD never exceeded 30 individuals, for a combined total of slightly more than 200 cases. Four patients developed vCJD several years after receiving blood that had not been leukocyte depleted from donors who had later succumbed to vCJD. However, whether these cases were transfusion-transmitted is by no means self-evident [9], and non-leukocyte depleted blood is no longer used in most countries. The indefinite deferral of donors who spent > 6 months in the UK during the BSE era has likely cost us, and continues to cost us, millions of units of, most likely, perfectly safe blood. Similarly, changes in eligibility for donors with a history of travel, surgical interventions or taking medications could likewise be imagined without risking adverse effects on transfusion safety. Thus, certain current exclusion criteria can probably be relaxed without sacrificing the (perceived) safety of blood to which society has grown accustomed.

In addition to loss of donations because of deferrals, the other reason for the shrinking blood supply is the decreasing willingness of young people to donate blood. Fewer than 5% of the presumably eligible donors actually donate blood, so the untapped population of suitable blood donors is huge [8]. Thus, instead of relaxing donor eligibility criteria, more imaginative new donor recruitment, better public awareness and better political/societal support of blood donation could solve our problem without jeopardizing, or appearing to jeopardize (as in the case of re-admission of non-practicing MSM), the standard of blood safety the public expects.

### How safe is our blood?

A basic principle of medical treatment is the Hippocratic oath "primum nihil nocere" (Latin: First, do no harm). This principle can be stifling in situations where harms and benefits must be weighed against one another. As a biological substance from a particularly poorly controlled source, blood has always been an obvious way in which pathogens can easily be transmitted. However, screening methods have become available and have steadily improved blood safety. First, serological assays became available, which assessed the immunological response of the host to an infectious agent, and were later supplemented with NAT tests for the infectious agent itself. Much of the effort to develop more sensitive infectious disease marker (IDM) screening methods was fuelled by the AIDS scandal in the 80s and 90s, when many patients were infected and killed by HIV transmitted through contaminated blood products [10-15]. Germany was among the first to implement NAT for all blood donations for hepatitis B, hepatitis C and HIV-1 [16,17]. Similar technology is now in use in one-third of all countries around the world [18]. A total of 300 million blood donations later, 244, 680 and 1,884 NAT-only positive blood donations with HIV-1, HCV and HBV, respectively, have been identified; each likely stands for an averted infection [18]. NAT technology has shrunk the diagnostic window between infection and detection to a minimum (< 10 days for HiV and HCV, 30 days for HBV). The residual risk for HCV, HIV-1 and HBV in Germany was recently estimated as 1 per 10.88, 4.3 and 0.36 million transfusions, respectively [19]. The power of NAT is particularly apparent in places like South Africa with its high prevalence of blood-borne infections, where introduction of NAT managed to control the risk of transfusion-transmitted virus infection that had been expected to rise dramatically after inclusion of donors from high-risk populations [20]. Nevertheless, NAT is more sensitive to subtle changes of the viruses than serology; mutations within primer and probe binding regions led to three false-negative NAT results in Germany in 2010 alone [21]. As a consequence, the federal authority (Paul-Ehrlich-Institute) recently recommended dual-targeting, that is, amplification in two conserved genome regions, for HIV-1 [21]. An alternative or complementary strategy to improve blood safety might be implementation of fourth generation antigen-antibody assays, which are available for HIV-1 with others to follow suit. The diagnostic window period will likely be similar to that of NAT. Emerging pathogens like dengue, Chikungunya and hepatitis E virus or malaria will present future challenges for blood transfusion services.

As an alternative to ever more sensitive test methods and ever broader pathogen spectra, global pathogen inactivation could be implemented. However, the need for different technologies for the different blood components, price, concerns about cell quality, long-term recipient safety, loss of product and other disadvantages have thus far impeded wide-spread use of available and emerging pathogen inactivation technologies.

Because of the sensitive screening methods used today, blood has become a relatively safe drug. Nevertheless, it should be self-intuitive that any relaxation of donor deferral rules which allow populations with an increased risk for certain transmissible diseases to donate blood, will be associated with an increased risk for recipients. It is always possible, as we have seen, to miss infectious agents [21], and a certain window period, although short, remains. Since relaxation of deferral criteria has not often been employed, (the South African example given above [20] being a unique exception), and numbers for at-risk individuals wishing to become blood donors are unknown, useful estimates of the risk increase can not be provided.

## Conclusions

Does a society have the right to expect a maximally safe blood supply if that society is not willing to reciprocate by donating in adequate quantity? A host of data suggests that in the very near future with our current strategies we may not be able to satisfy the need for blood, in significant part because we are failing to recruit new blood donors from the vast majority of people who choose not to donate blood even though they would be eligible by current standards. The alternative to new donor recruitment would be to relax current eligibility criteria, which may impact upon safety by allowing some permanently deferred populations to donate blood at the potential risk of jeopardizing the safety of blood. To a degree, that is what the recent decision in the UK to allow non-practicing MSM to donate blood is doing. However, we predict that this specific decision will not have (tangible) adverse effects on blood safety, although there may be some changes in perceived safety. That rule change should be seen as a political move, a half-hearted political nod to MSM, probably partly in response to student unions' threats to boycott blood donor drives in universities unless "discrimination" of MSM by blood services is discontinued. On its own, the change will likely only be consequential for blood supply or blood safety in that it avoids the threatened student boycott and prevents the loss of those coveted donors. At the same time, though, it may adversely affect the public's perception of blood safety. Only if we can motivate new volunteers to become donors can we avert the impending public loss of faith in blood products, and meet the ever-increasing need for these products.

## List of abbreviations

BSE: Bovine Spongiform Encephalopathy; HBV: hepatitis B virus; HIV: human immunodeficiency virus; IDM: infectious disease marker; MSM: men who have sex with men; NAT: nucleic acid testing; doing PCR for viral DNA/RNA; vCJD: Creutzfeld-Jacob's Disease-new variant, mad cow disease

## Competing interests

The authors declare that they have no competing interests.

## Authors' contributions

HB, MS, KH, JS and ES co-wrote the manuscript. All authors read and approved the final manuscript.

## Pre-publication history

The pre-publication history for this paper can be accessed here:

http://www.biomedcentral.com/1741-7015/10/29/prepub
